# Rehabilitation of patients in the subacute phase of stroke using the ReHand robotic system: a randomized controlled trial

**DOI:** 10.3389/fnhum.2025.1690643

**Published:** 2025-11-24

**Authors:** Mariyam Amirbekova, Tokzhan Kispayeva, Akmaral Izbassarova, Ausra Adomaviciene, Marina Sorokina, Tomiris Zhunussova

**Affiliations:** 1 Institute of Life Sciences, Karaganda Medical University, Karaganda, Kazakhstan; 2 School of Nursing Education, Karaganda Medical University, Karaganda, Kazakhstan; 3 Department of Physical Medicine and Rehabilitation, Sports Medicine, Kazakh National Medical University Named After S.D. Asfendiyarov, Almaty, Kazakhstan; 4 Department of Rehabilitation, Physical and Sports Medicine, Faculty of Medicine, Vilnius University, Vilnius, Lithuania; 5 Department of Informatics and Biostatistics, Karaganda Medical University, Karaganda, Kazakhstan; 6 School of Medicine, Karaganda Medical University, Karaganda, Kazakhstan

**Keywords:** stroke, upper limb, robotic therapy, soft exoskeleton, rehabilitation, neuroplasticity

## Abstract

**Background:**

Upper limb motor impairment after stroke is a major cause of limitations in daily activities and reduced quality of life. Although traditional rehabilitation is effective, it is often insufficiently intensive and lacks focus on fine motor activation. Portable soft exoskeletons offer a promising approach to intensify recovery. This trial aimed to evaluate the effectiveness of the ReHand robotic system in subacute stroke rehabilitation.

**Methods:**

This Randomized Controlled Trial (RCT) included 120 patients in the subacute period of stroke. Participants were stratified by age and motor deficit severity and randomized into the intervention group (robotic therapy + standard therapy) or control group (standard therapy only). The intervention was delivered 5 times per week over 8 weeks. The primary outcome was change in Fugl-Meyer Assessment for Upper Extremity (FMA-UE); secondary outcomes included Barthel Index (BI), Functional Independence Measure (FIM), National Institutes of Health Stroke Scale (NIHSS), modified Wolf Motor Function Test (mWMFT), Frenchay Arm Test (FAT), Disabilities of the Arm, Shoulder and Hand (DASH), and Hospital Anxiety and Depression Scale (HADS). Statistical analysis was performed in Python (v3.11). Normality was assessed with the Shapiro–Wilk test; the Mann–Whitney test was used for intergroup comparisons; Pearson’s χ^2^ test with Yates’ correction was used for categorical variables. Effect size was calculated using Cliff’s delta; rank-ANCOVA was performed via the Quade method. Significance was set at *p* < 0.05.

**Results:**

The intervention group demonstrated significantly greater improvements in upper limb motor impairment compared to the control group, as reflected by larger gains in FMA-UE scores. Clinically meaningful improvements (≥5.25 points) were observed in 91.7% of patients in the intervention group versus 43.3% in the control group. All secondary outcomes also showed significant improvements in the intervention group (*p* < 0.001). No adverse events were reported in either group.

**Conclusion:**

When combined with standard therapy, the ReHand robotic system may enhance upper limb recovery after stroke and appears to be a safe and feasible adjunct to multidisciplinary rehabilitation programs.

**Clinical trial registration:**

ClinicalTrials.gov, identifier (NCT06937346).

## 1 Introduction

Stroke remains one of the leading causes of adult disability worldwide, resulting from the sudden interruption of cerebral blood flow and subsequent neuronal death. The resulting motor, sensory, and cognitive deficits impose a substantial socioeconomic burden on healthcare systems and rehabilitation services. Given that upper-limb impairment affects up to 80% of stroke survivors and significantly limits independence, optimizing post-stroke rehabilitation remains a major clinical priority. With an aging population and widespread vascular risk factors, over 12 million new stroke cases are reported annually. Therefore, the prevention of primary and recurrent stroke, along with effective rehabilitation, remains a strategic global health priority. Early detection and management of risk factors can reduce stroke incidence. Nevertheless, despite advances in prevention, a large proportion of patients still require long-term, high-quality rehabilitation, particularly for upper limb recovery. Many experience impaired fine motor skills of the hand, including difficulties with precise and coordinated finger movements, grasping, and object manipulation (GBD 2019 Stroke Collaborators, 2021; Rakhimova et al., 2021; Amirbekova and Kispayeva, 2024; Kispaeva et al., 2025; Purrahman et al., 2023; French et al., 2016). These limitations significantly reduce independence in everyday life: from buttoning clothes to using cutlery and a smartphone (Alsubiheen et al., 2022).

Thus, the restoration of fine motor skills is a priority task of post-stroke rehabilitation. However, traditional approaches such as therapeutic exercise, occupational therapy and mirror therapy require significant resources, despite their proven effectiveness. Often, they do not provide sufficient intensity, especially in an outpatient setting (Kispayeva et al., 2025; Amirbekova et al., 2025).

Modern data emphasize the importance of intensive, repetitive and targeted therapy to stimulate brain neuroplasticity in the subacute and chronic phases (Tseng et al., 2024). In this context, robotic technologies offer new opportunities. They provide precise, repeated movements, adaptation to the patient’s capabilities and the possibility of home use (Shi et al., 2021). Among robotic solutions, special attention is paid to soft wearable gloves. They provide repeated and controlled movements taking into account the functional state of the patient (Radder et al., 2019; Lim et al., 2023; Hernández Echarren and Sánchez Cabeza, 2023). Such technologies increase the accuracy of exercises and promote patient involvement, stimulating both motor and sensory functions (Fiska et al., 2025). Despite the active development of this technology, there is a lack of clinical studies aimed at restoring fine motor skills. This is especially true for randomized controlled trials using objective scales for assessing functional changes (Chen et al., 2023; Prange-Lasonder et al., 2017).

A recent randomized controlled trial (RCT) showed that the use of a soft glove produced comparable results with repetitive Transcranial Magnetic Stimulation (rTMS) on the Fugl-Meyer Assessment for Upper Extremity (FMA-UE) scale in patients with severe motor impairments. Although no significant difference was found in the Barthel Index, these data support the potential of gloves as an effective alternative (Wang et al., 2022). A large meta-analysis demonstrated that soft robotic gloves, when used in combination with conventional therapy, resulted in greater improvements in upper limb motor function compared with conventional therapy alone, including both proximal and distal segments, as well as improving functional task performance (Ko et al., 2023b). Additional studies confirmed clinical benefits in the form of increased grip strength, dexterity, and Action Research Arm Test (ARAT) and FMA-UE scores, especially in patients with mild spasticity. Moreover, the interventions were safe and did not increase spasticity (Hsu et al., 2024; Coskunsu et al., 2022; Park et al., 2020). However, the effectiveness depends on the time since stroke, the level of residual spasticity and the number of sessions. Studies with exoskeletons demonstrate positive neuronal effects, including increased cortical excitability and improved kinematic movement parameters. The subacute phase of stroke is characterized by heightened neural plasticity and responsiveness to motor training intensity. Evidence suggests that rehabilitation delivered during this window, when the brain exhibits increased capacity for cortical reorganization, can maximize motor recovery outcomes. However, few studies have systematically investigated how the dose and intensity of upper-limb training influence neuroplastic mechanisms during this critical phase and treatment parameters and choice of movement pattern require further study (Singh et al., 2021; Akgün et al., 2024; Ko et al., 2023a). Systematic reviews confirm a dose-dependent effect for sessions longer than 30 min, especially in the chronic phase of stroke (Ko et al., 2023b). However, many studies suffer from limited sample sizes, high methodological heterogeneity, and lack of standardized approaches, which makes it difficult to generalize the results (Chen et al., 2023; Prange-Lasonder et al., 2017).

Against the background of these limitations, soft robotic gloves represent a promising direction. Simplicity of their design, autonomy and the possibility of a repeatable mode, in which the healthy hand controls the movement of the affected one, are important advantages. They allow activating interhemispheric connections and promoting the reduction of motor impairment through synchronous, symmetrical movements.

Recent investigations by Cantillo-Negrete and colleagues have further explored the integration of robotic systems with neural interfaces for upper-limb rehabilitation. In their ReHand-BCI randomized controlled trial, the combination of a soft robotic glove and brain–computer interface enhanced hand motor recovery compared with conventional therapy. Similarly, their earlier crossover feasibility study demonstrated that coupling a robotic hand orthosis with BCI feedback facilitated task-specific cortical activation and motor improvement in stroke survivors (Cantillo-Negrete et al., 2025; Cantillo-Negrete et al., 2021).

The ReHand system is a technology that integrates portability with two-way sensor control, allowing users to replicate the movements of a healthy hand onto a paretic (weakened) hand in real-time using signals from sensors embedded in a lightweight exoskeleton glove. This innovative approach enables intensive individual training, both at home and in clinical settings, helping to reduce motor impairment through repetitive movements.

Unlike many existing systems, ReHand allows users to reconfigure their functions without relying on external power supplies or computers, providing various training modes tailored to the patient’s specific needs and abilities. This flexibility makes it a valuable tool for rehabilitation and recovery from stroke.

Most existing devices remain bulky and require external power or connection to a computer. In addition, they are focused on passive mobilization, which limits their use at home (Yap et al., 2017). At the same time, a number of studies are characterized by a short duration of therapy and a limited number of participants. These factors complicate a reliable assessment of sustainability and reproducibility of clinical effects (Proulx et al., 2020).

Thus, portable and affordable devices aimed at symmetrical motor activation and regular movement repetition represent a promising direction (Prange-Lasonder et al., 2022). However, they require further clinical validation to confirm their effectiveness and possibility of integration into personalized rehabilitation programs.

The aim of this randomized controlled trial is to evaluate the effectiveness of the ReHand portable robotic system in the rehabilitation of patients after stroke.

We hypothesized that adding the ReHand robotic therapy to standard rehabilitation would lead to greater improvements in upper-limb motor function, compared with standard therapy alone. Secondary hypotheses were that ReHand-assisted therapy would yield additional benefits in functional independence and psychosocial outcomes.

## 2 Materials and methods

### 2.1 Trial design

This clinical trial is single-center, randomized and controlled, with parallel groups and single-blinding (assessor and statistician masking). Participants were randomized in a 1:1 ratio to the main and control groups. The RCT was designed and described in accordance with the CONSORT 2025 guidelines (Hopewell et al., 2025). To reduce the risk of bias, stratified randomization was used by two factors: age according to the World Health Organization (WHO) classification (18–59 years – young and middle age and 60–74 years – old age) and the baseline level of upper limb motor deficit assessed by the FMA-UE scale (21–50 points - moderate and 51–66 - mild) (Prokopenko et al., 2016). The number of participants was equal in each stratum.

The trial was conducted at the Neuron Rehabilitation Center in the cities of Karaganda and Astana, Kazakhstan, from March 2024 to May 2025. The study was conducted in accordance with the Declaration of Helsinki, and approved by the Local Ethics Committee of the Karaganda Medical University (Protocol No. 3, dated February 27, 2024). The trial protocol was registered in the international clinical trials database ClinicalTrials.gov under the identifier NCT06937346.

### 2.2 Participants

In the period from March to May 2024, patients in the subacute phase of stroke meeting the inclusion criteria for the trial were recruited at an outpatient appointment at the Neuron rehabilitation center (Karaganda and Astana cities).

Inclusion criteria: age 18 years and older; first-ever diagnosed ischemic or hemorrhagic stroke in the subacute phase, confirmed by Computed Tomography (CT) or Magnetic Resonance Imaging (MRI); motor impairment of the upper limb according to the FMA-UE scale > 20 points; clear consciousness; no cognitive impairment (according to the Mini-Mental State Examination (MMSE) scale ≥ 24 points) that make it difficult to understand and follow instructions; no somatic conditions that could interfere with the use of hand exoskeleton system (such as rheumatoid arthritis, severe muscle hypertonicity, joint pathology or fractures); the presence of upper limb and fine motor skills disorders; presence of written informed consent.

Exclusion criteria: history of recurrent stroke; rheumatological diseases; problems (including contractures and severe pain syndromes) that could interfere with participation; frequently recurring or profuse bleeding of various origins; febrile fever or subfebrile fever of unknown origin; acute infectious diseases; acute osteomyelitis; acute deep vein thrombosis; complicated cardiac arrhythmias or heart failure according to functional class IV by classification of the New York Heart Association (NYHA); active stage of all forms of tuberculosis; malignant neoplasms (clinical group IV); respiratory failure of grade III or higher; various purulent (pulmonary) diseases with significant intoxication; diseases in the decompensation stage, namely, uncorrectable metabolic diseases (diabetes mellitus, myxedema, thyrotoxicosis and others); functional insufficiency of the liver or pancreas of the III degree; epilepsy in the attack period; severe psychiatric disorders (e.g., psychosis, major personality disorders, or behavioral dysregulation); purulent skin diseases and contagious skin diseases (scabies, fungal diseases and others); anemia of the 2–3 degree; dystrophy of the 3 degree in the presence of other concomitant diseases that prevent active participation in the medical rehabilitation program for 2–3 h a day.

All participants were informed in advance about the aims and procedures of participation. Written informed consent was obtained before any study procedures, including baseline assessment (T0) and initiation of the intervention. The trial was conducted in accordance with the principles of confidentiality: personal data were coded and stored in encrypted form, accessible only to the research team. Participants could refuse participation at any stage without consequences for further treatment.

The safety of intervention was monitored throughout the trial. All adverse events were recorded. Monitoring data were stored in accordance with regulatory requirements.

### 2.3 Randomization and allocation

One hundred and twenty patients were randomized and divided into two groups of sixty people: the intervention group received combined rehabilitation using the ReHand robotic system and standard therapy; the control group received standard therapy only. The total therapy period for each patient was 60 days. The assessment was carried out at two time points: before the start of therapy (T0) and after its completion (T1).

Stratified block randomization was used to minimize systematic error and ensure a balanced distribution of participants. Stratification was carried out according to two clinically significant characteristics: age and the baseline severity of motor impairment of the upper limb according to the FMA-UE scale (Prokopenko et al., 2016). Thus, four strata were formed. Within each stratum, a separate block randomization was carried out with an equal distribution of participants between the groups.

The random sequence was generated using Random Allocation Software (version 1.0). Randomization parameters: method - stratified block randomization; allocation ratio - 1:1 between groups (robotic therapy with ReHand + standard therapy and standard therapy only); block allocation - balanced.

The completed sequence was exported to a password-protected Excel file and managed by an independent coordinator not involved in the intervention or outcome assessment.

Participants were allocated centrally at the time of inclusion in the trial. After signing the informed consent and passing the baseline assessment (T0), the researcher contacted the coordinator, who provided the group code based on the table.

Allocation concealment was ensured by the fact that the researcher did not have access to the randomization table and could not predict the patient’s belonging to one or another group until randomization.

### 2.4 Blinding

The trial used single-blinding at the level of assessors and statisticians. The specialists who performed the clinical assessment of the results at the baseline (T0) and after the therapy (T1) were blinded to the allocation of participants to groups. Statistical analysis was also performed by an independent specialist blinded to the allocation of participants to therapy groups, which ensured additional objectivity in the interpretation of the results.

Patients were not blinded for ethical reasons and also because of the obvious differences between the interventions (combination therapy using a robotic system + standard therapy versus standard therapy only), which made double blinding impossible.

Assessor blinding was ensured by delineating functions between members of the research team. The staff who performed the therapeutic intervention were not involved in data collection and analysis. All assessments were performed by independent specialists who did not have access to information about the group allocation of the participants.

The statistician was provided with a de-identified database, in which the groups were designated by neutral codes (“Group A” and “Group B”), without indicating the type of treatment. This allowed us to minimize the risk of bias in the analysis of the results.

### 2.5 Interventions

Participants randomized to the intervention group received a combined intervention including robotic therapy with the ReHand system and a standard rehabilitation program. Participants in the control group received only standard rehabilitation, without robotic technologies.

#### 2.5.1 ReHand robotic therapy

ReHand is a software and hardware robotic complex designed to reduce upper limb motor impairment in patients after stroke. The device functions as a hand exoskeleton, providing passive training of the paralyzed fingers on the affected hand.

The key feature of the system is the use of an innovative method of repeating movements: the movements of the patient’s healthy hand are read using a sensor glove worn on it and transmitted in real time to the rehabilitation glove worn on the affected upper limb. This allows the affected hand to accurately repeat the movements of the healthy one, thereby activating neural mechanisms and promoting the restoration of motor function.

The device includes mechanized elements that provide passive mobilization of finger and hand joints. The system allows for training individualization, automatically adapting the intensity, amplitude and nature of movements to the patient’s current functional capabilities. Several training modes are provided: from simple repetitive movements to complex functional tasks with increasing load.

**Figure 1:**
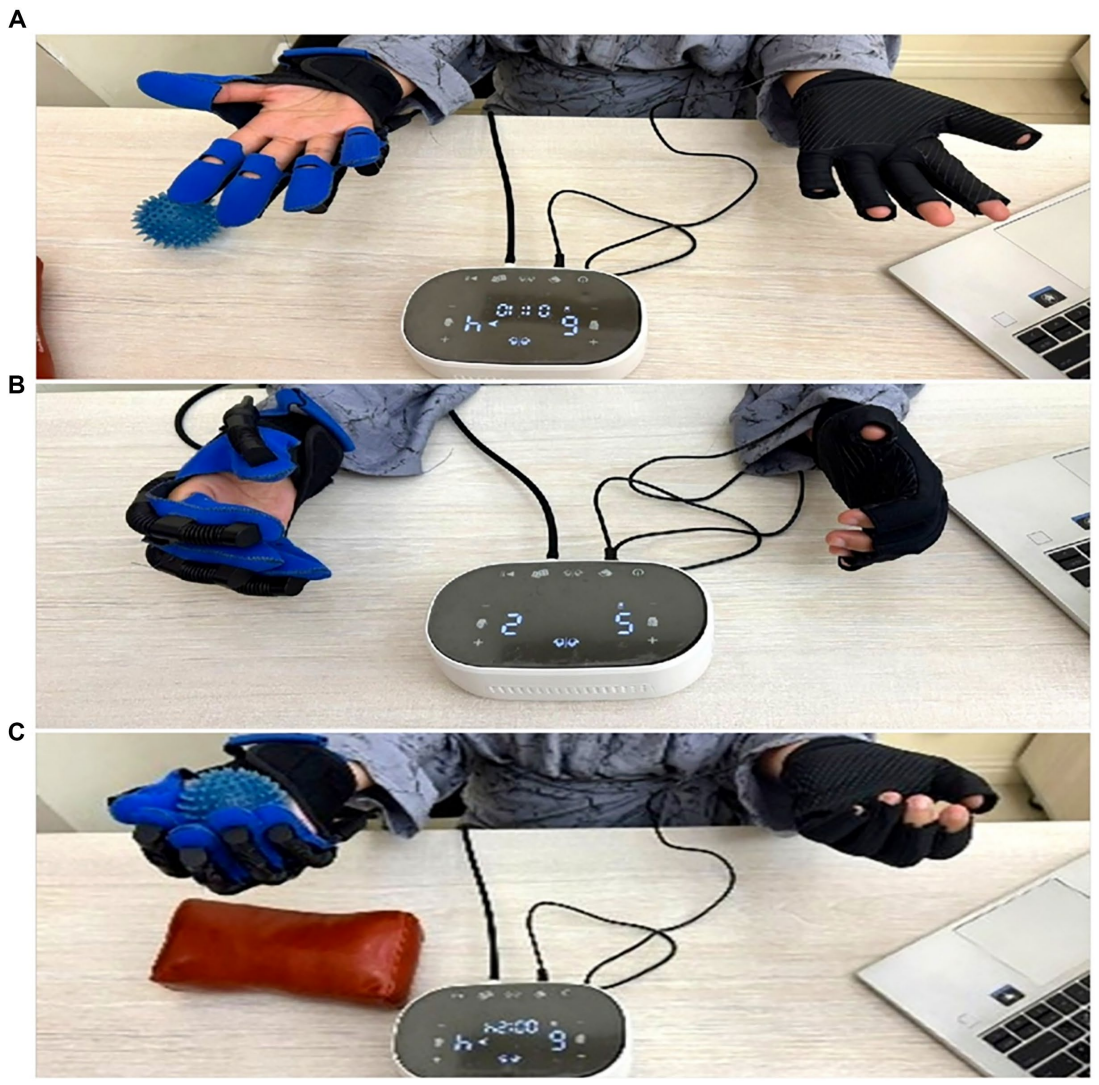
Key components and operating principles of the ReHand robotic rehabilitation system for upper limb recovery after stroke. **(A)** Robotic-assisted hand opening: the system facilitates finger extension and release of a clenched fist to reduce flexor spasticity and enhance voluntary hand opening. **(B)** Grasp training: the patient performs a controlled gripping task with assistance from the robotic glove, aimed at improving flexor strength and coordination. **(C)** Functional training: the patient manipulates objects simulating activities of daily living, targeting improvements in fine motor control and task-specific hand function with the robotic glove.

The intervention protocol in the intervention group included 5 sessions per week, 45 min each, for 8 weeks. Each session included active work with the ReHand system in combination with passive exercises under the supervision of a specialist. The exercises were aimed at improving joint mobility, developing grip accuracy, strength and coordination. The load was increased gradually, taking into account the individual progress of the patient. All procedures were performed on an outpatient basis by a trained specialist (Figure 1).

#### 2.5.2 Standard rehabilitation

Standard rehabilitation received by all participants (both in the main and control groups) was a comprehensive program aimed at restoring the motor impairment of the upper limb. The therapy included exercises for increasing the range of active and passive movements in joints, muscle strength and endurance, and improving the precision, smoothness, and coordination of movements of the affected limb (Amirbekova and Kispayeva, 2024). The therapy was based on the principles of functional focus, high repetition, gradual complication of tasks, and inclusion in everyday activities. Exercises were selected individually, taking into account the motor deficit, cognitive status, and clinical tasks. All interventions corresponded to modern principles of neuroplasticity: adequate intensity, task specificity, motivational significance, and functional focus. Patients were provided with feedback on the progress of task completion, and active participation in therapy was encouraged.

Tolerability and safety were monitored at each session. The patient’s adherence to treatment, level of fatigue, pain syndrome and concomitant complications affecting participation in therapy were monitored. Exercises were selected individually according to each patient’s motor deficit and rehabilitation goals, focusing on grasp–release tasks, reaching, and object manipulation relevant to daily activities. Physiotherapists provided both *knowledge of performance* (verbal correction of movement quality) and *knowledge of results* (feedback on task completion and accuracy). Fatigue and pain levels were monitored at the end of each session using a 10-point numeric rating scale (0 = none, 10 = maximum). Sessions were paused or modified if fatigue or pain exceeded 7 points.

Standard rehabilitation was carried out according to a similar principle: five 45-min sessions per week for 8 weeks in an outpatient setting and under the supervision of experienced specialists.

Thus, the main difference between the groups was the addition of robotic therapy ReHand in the intervention group, along with identical duration, frequency and basic content of standard therapy.

### 2.6 Outcome measures

The effectiveness of the intervention was assessed twice: at baseline and after completion of the 8-week program. The main scale for assessing motor impairment was the FMA-UE. Additional scales were used to comprehensively characterize the patients’ condition, including functional, psychoemotional and neurological parameters.

#### 2.6.1 Primary outcome

The motor function of the upper limb was assessed using the FMA-UE scale, one of the most widely used validated instruments for assessing motor impairment after stroke. The maximum score is 66, where higher values reflect a better state of the motor impairment of the upper limb. This method is standardized, sensitive to changes in the dynamics of recovery and recommended for clinical studies and practice (Hernández et al., 2019). The minimal clinically important difference (MCID) on the FMA-UE scale is 5.25 points (Hiragami et al., 2019).

#### 2.6.2 Secondary outcomes

For a comprehensive assessment of the clinical condition of patients after stroke, along with the primary FMA-UE scale, the following validated scales were used, reflecting the level of functional independence, neurological deficit, psychoemotional state, as well as the quality of motor task performance and subjective functionality of the upper limb.

To assess the overall level of functional independence of patients, the Functional Independence Measure (FIM) scale was used - a standardized and validated tool widely used in rehabilitation practice. The overall total score ranges from 18 to 126, with higher values reflecting a higher level of functional independence. The scale covers such aspects as self-care, sphincter control, movement, communication and social cognition. FIM allows assessing the dynamics of recovery during rehabilitation and is a recommended tool in international rehabilitation programs and clinical trials (Gkouma et al., 2022).

The functional state of the upper limb was assessed using the Frenchay Arm Test (FAT), a validated and reliable tool for assessing voluntary motor function of the paretic arm after stroke. The test is easy to use, does not require complex equipment, and is widely used both in clinical practice and in scientific research to assess the effectiveness of rehabilitation interventions (Laclergue et al., 2023). To assess the motor function of the upper limb, we used the Wolf Motor Function Test (WMFT), a validated tool used in clinical practice and scientific research to assess restored motor activity after stroke. This trial had a modified version of the test with an assessment of only the quality of task performance, without taking into account the time. The maximum score is 75, where higher values reflect better quality of motor function. This version of the Modified Wolf Motor Function Test mWMFT is sensitive to changes in the recovery and is recommended for assessing the effectiveness of rehabilitation interventions (Turtle et al., 2020).

Both the FAT and the mWMFT were included to provide complementary perspectives on upper-limb performance. While the FAT captures the ability to perform discrete functional tasks reflecting real-world use, the mWMFT assesses the quality and control of movement during standardized motor activities. Including both scales allowed us to capture task performance and movement quality as distinct but related aspects of recovery.

The National Institutes of Health Stroke Scale (NIHSS) was used to assess the degree of neurological deficit in patients. This scale is a standardized clinical tool used to assess the severity of stroke. The total score ranges from 0 to 42, where higher values indicate more severe neurological deficit. The NIHSS is widely used at all stages of stroke treatment and rehabilitation, from the acute period to long-term follow-up. It is validated, reproducible, and sensitive to changes in the patient’s condition, which makes it useful both in clinical practice and in scientific research to assess the effectiveness of therapy (Cummock et al., 2023).

The Barthel Index (BI) was used to assess patients’ ability to independently perform daily activities. It is designed to assess the patient’s dependence on outside assistance in daily life, such as eating, dressing, hygiene, moving, ambulating, and monitoring physiological functions. BI is a validated and sensitive tool for assessing the effectiveness of rehabilitation and is widely used both in clinical practice and in research (Watanabe et al., 2024).

The Disabilities of the Arm, Shoulder and Hand (DASH) questionnaire was used to assess patients’ subjective perception of limitations in daily activities related to upper limb function. This is a validated self-assessment tool designed to assess physical dysfunction and symptom severity in the shoulder, arm and hand. The final score is calculated using a special formula and ranges from 0 to 100 points. In this case, 0 points correspond to the absence of limitations, and 100 points correspond to maximum difficulties and severe symptoms. The questionnaire is sensitive to clinically significant changes and is widely used to assess the effectiveness of rehabilitation interventions for upper limb pathology, including conditions after stroke (Brindisino et al., 2024).

The Hospital Anxiety and Depression Scale (HADS) was used to assess the psychoemotional state of patients. This is a validated and widely used self-assessment tool designed to identify symptoms of emotional distress in patients with somatic diseases, including stroke. The overall score for the entire scale ranges from 0 to 42 points. Higher values indicate more pronounced symptoms of anxiety and/or depression. HADS is sensitive to changes in the psychoemotional state and is recommended for monitoring the emotional background during rehabilitation after stroke (Karlsson et al., 2024).

### 2.7 Sample size calculation and statistical methods

The sample size was calculated using G*Power 3.1.9.7 software based on the primary variable, the FMA-UE score. According to published data, a clinically significant difference between groups is at least 5 points, the standard deviation is about 9 points, which is equivalent to the expected effect of Cohen’s d = 0.58 (Winters et al., 2016; Lundquist and Maribo, 2017; Huynh et al., 2023). To achieve a power of 80% (1–*β* = 0.80) at a significance level of *α* = 0.05 (two-sided test), the minimum required number of participants in each group was 48 people. Taking into account possible losses of up to 25%, the final sample was increased to 120 participants (60 in each group).

Data analysis was performed using Python programming language (version 3.11). Categorical variables (gender and stroke type) were encoded using the one-hot encoding method. Numerical predictors (age and number of days after stroke) were standardized using StandardScaler. The analysis was performed according to the intention-to-treat principle.

Non-parametric statistics were applied because most continuous variables did not follow a normal distribution. Effect sizes were calculated to estimate the magnitude of differences rather than relying solely on *p* values.

The normality of the distribution of continuous variables was tested using the Shapiro–Wilk test. In the absence of a normal distribution, the data were described as the median (Q1; Q3). Categorical variables are presented as absolute and relative frequencies (%), with 95% confidence intervals (CI) calculated for proportions.

Between-group differences in changes in values were assessed using the Mann–Whitney test. Pearson’s χ^2^ test with Yates’ correction was used to analyze categorical variables. All statistical tests were two-sided. The significance level was set at *p* < 0.05. The Cliff’s Delta coefficient was used to assess the effect size when comparing differences (deltas) between groups.

As an additional analysis confirming the reliability of the results, rank-based ANCOVA was performed using the Quade method, taking into account the initial values as covariates.

## 3 Results

### 3.1 Participants

**Figure 2:**
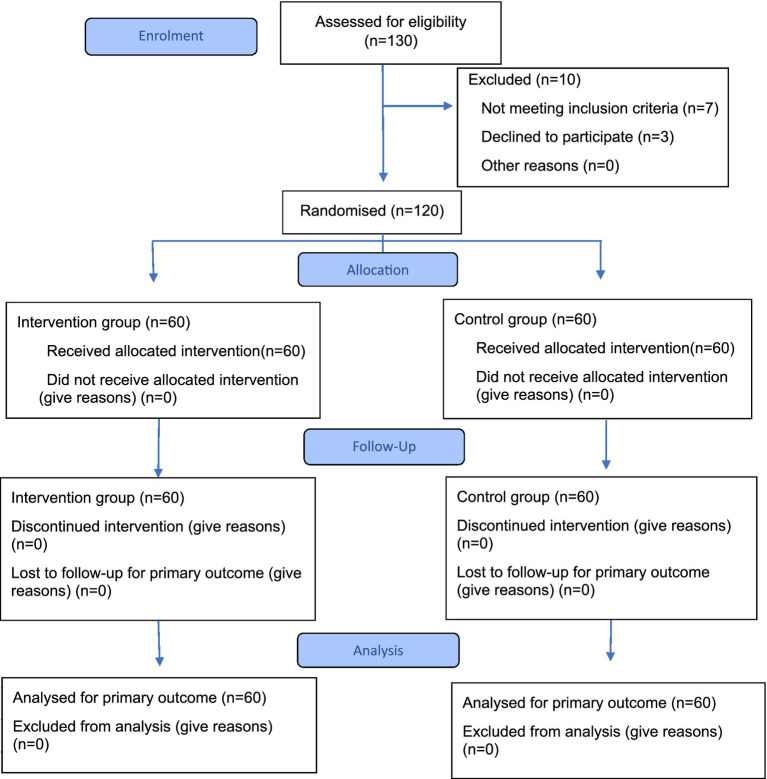
CONSORT 2025 flow diagram of the progress through the phases of a randomized trial of two groups (that is, enrolment, intervention allocation, follow-up, and data analysis).

From March 2024 to May 2024, 130 patients with upper limb motor impairment after stroke were screened at the Neuron rehabilitation center in Karaganda and Astana cities, Kazakhstan. Of these, 120 met the inclusion criteria and were randomized. After stratified randomization, 60 participants were assigned to the intervention group and 60 to the control group. All participants completed the intervention and were re-evaluated after 8 weeks. No loss in the sample was recorded (Figure 2).

### 3.2 Demographic and clinical characteristics of participants

At baseline, demographic and clinical characteristics of participants were collected, including age, gender, stroke type (ischemic or hemorrhagic), number of days since stroke, and scale scores. The primary outcome measure was the FMA-UE scale. Secondary outcomes included BI, FIM, NIHSS, FAT, mWMFT, DASH, and HADS.

Comparison of baseline characteristics between groups revealed no statistically significant differences. Mean age, gender, stroke type, and time since stroke were comparable (*p* > 0.05) for all measures. These data confirm successful randomization and comparability of the groups at baseline.

The results of the assessment of demographic and clinical characteristics of participants in the main and control groups at baseline are presented in Table 1.

**Table 1:** Baseline demographic and clinical characteristics of participants.

Characteristic	Intervention group (*n* = 60)	Control group (*n* = 60)	*p*-value
Age(years), mean ± SD	59.7 ± 9.0	59.3 ± 10.0	0.84
Sex, *n* (%) [95% CI]			0.83
Male	46 (76.7%)	44 (73.3%)	
Female	14 (23.3%)	16 (26.7%)	
Type of stroke, *n* (%) [95% CI]			1.0
Ischemic	44 (73.3%)	45 (75.0%)	
Hemorrhagic	16 (26.7%)	15 (25.0%)	
Number of days after stroke, mean ± SD	104.2 ± 45.0	106.0 ± 46.7	0.88
Primary outcome, median (Q1; Q3)			
FMA-UE	29.0 (24.8; 32.0)	29.0 (26.0; 32.0)	0.86
Secondary outcomes, median (Q1; Q3)			
BI	40.0 (35.0; 40.0)	45.0 (40.0; 50.0)	0.0
NIHSS	8.0 (7.0; 11.0)	8.0 (5.8; 11.0)	0.78
FIM	52.0 (50.0; 55.2)	54.5 (52.0; 58.0)	0.009
FAT	1.0 (0.0; 1.0)	1.0 (0.0; 1.0)	0.83
mWMFT	36.0 (33.0; 39.0)	37.0 (34.0; 40.0)	0.06
DASH	46.0 (43.0; 48.0)	46.5 (44.0; 49.2)	0.11
HADS	28.0 (25.0; 33.0)	31.0 (28.0; 34.0)	0.029

FMA-UE, Fugl-Meyer Assessment for Upper Extremity; BI, Barthel Index; NIHSS — National Institutes of Health Stroke Scale; FIM, Functional Independence Measure; FAT, Frenchay Arm Test; mWMFT, Modified Wolf Motor Function Test; DASH, Disabilities of the Arm, Shoulder and Hand questionnaire; HADS, Hospital Anxiety and Depression Scale. Continuous variables are presented as mean ± standard deviation (SD) or median (Q1; Q3), and categorical variables as *n* (%) with 95% confidence intervals (CIs). Between-group comparisons were conducted using the Mann–Whitney U test for non-normally distributed continuous variables and the chi-squared (χ^2^) test with Yates’ correction for categorical variables. A *p-*value < 0.05 was considered statistically significant.

### 3.3 Comparison of groups by baseline clinical parameters

Comparison of FMA-UE values between groups at baseline showed no statistically significant differences (*p* = 0.86), confirming the comparability of the groups according to the primary outcome measure.

Analysis of secondary indicators revealed higher baseline values of FIM and BI in the control group (*p* < 0.05), which may indicate higher functional independence in control group. Also, the control group had higher scores of anxiety and depression according to the HADS scale (*p* = 0.02). At the same time, the differences in NIHSS, FAT, mWMFT and DASH were statistically insignificant (*p* > 0.05), indicating comparable neurological and functional status of the upper limb.

Due to the identified baseline differences in a number of parameters, the further analysis included a covariance adjustment (rank ANCOVA), which provided a more accurate assessment of therapeutic effect.

### 3.4 Within-group changes in scale scores

Analysis of changes in clinical indicators over the 60-day intervention demonstrated a statistically significant advantage over the intervention group for all scales. The main improvement was observed compared to the control group on the FMA-UE scale, reflecting the restoration of motor impairment of the upper limb, with a pronounced effect of the intervention. MCID on the FMA-UE scale, set at 5.25 points (Hiragami et al., 2019), was achieved in 55 of 60 patients in the intervention group, which is 91.7%. In the control group, this improvement was recorded only in 26 of 60 participants (43.3%), which demonstrates the significant superiority of ReHand robotic therapy in the proportion of respondents who achieved the minimal clinically significant increase in upper limb function.

Among the secondary outcomes, significantly greater improvement in the intervention group was recorded in the following scales: functional independence (BI, FIM), reduction of neurological deficit (NIHSS), motor activity (mWMFT, FAT), as well as in the hand activity limitation index (DASH). In addition, a significant decrease of anxiety and depression according to the HADS scale was noted in the group receiving robotic therapy.

To account for the baseline differences observed in FIM, BI and HADS, a Quade rank ANCOVA was performed, adjusting for baseline values. The adjusted analysis confirmed that the between-group differences in changes remained statistically significant (*p* < 0.001 for both FIM, BI and HADS), supporting the robustness of the intervention effect.

These results confirm the effectiveness of intervention in relation to both motor and functional indicators, as well as the psychoemotional state of patients (Table 2).

**Table 2:** Between-group comparison of median differences (Δ) in clinical outcomes.

Measure	Intervention group, median (Q1; Q3)	Control group, *Δ* median (Q1; Q3)	*p*-value	Cliff’s delta (δ)
Primary outcome				
FMA-UE	16.5 (12.0; 20.5)	6.0 (3.0; 9.2)	0.0	0.721
Secondary outcomes				
BI	30.0 (25.0; 35.0)	10.0 (8.8; 15.0)	0.0	0.953
NIHSS	−4.0 (−5.0;-4.0)	−2.0 (−2.0; −1.0)	0.0	−0.741
FIM	22.5 (19.0; 28.0)	5.5 (3.0; 9.0)	0.0	0.940
FAT	2.0 (2.0; 3.0)	1.0 (0.0; 1.0)	0.0	0.798
mWMFT	18.5 (14.0; 23.2)	6.0 (4.0; 10.0)	0.0	0.817
DASH	−28.0 (−33.0; −23.0)	−7.0 (−11.2; −4.0)	0.0	−0.964
HADS	−13.0 (−15.0; −10.0)	−8.0 (−11.0; −5.8)	0.0	−0.528

Values represent the within-group change (Δ) from baseline to post-treatment, expressed as median (Q1; Q3). Between-group comparisons of these differences were performed using the Mann–Whitney U test with continuity correction. Effect sizes are presented as Cliff’s delta (δ). FMA-UE, Fugl-Meyer Assessment for Upper Extremity; BI, Barthel Index; NIHSS, National Institutes of Health Stroke Scale; FIM, Functional Independence Measure; FAT, Frenchay Arm Test; mWMFT, Modified Wolf Motor Function Test; DASH, Disabilities of the Arm, Shoulder and Hand questionnaire; HADS, Hospital Anxiety and Depression Scale. A *p*-value < 0.05 was considered statistically significant.

### 3.5 Sensitivity analysis

To test the stability of intergroup differences, a Quade rank analysis of covariance (rank ANCOVA) was performed, adjusting for baseline values for the BI, FIM, and HADS scales. In all cases, the differences between the groups in terms of change in the indicators remained statistically significant (*p* < 0.001), which confirms the stability of the results and reliability of the intervention effect.

These results confirm that the effect of robotic therapy is maintained after controlling for the baseline level, ensuring the reliability and interpretability of the observed differences.

### 3.6 Side effects

During the rehabilitation intervention, no cases of adverse effects were registered in the intervention group. All patients tolerated the robotic and standard therapy satisfactorily; there were no complaints of deterioration in condition, pain syndrome or other adverse reactions.

In the control group, one participant (*n* = 1; 1.66%) reported pain in the upper limb after standard rehabilitation. This episode was assessed as a temporary adverse event that did not require discontinuation of participation in the trial and did not affect the volume or duration of rehabilitation.

Thus, both types of intervention demonstrated a high safety profile and good tolerability, in the absence of serious adverse events.

## 4 Discussion

### 4.1 Key results

The data obtained suggest that combining ReHand robotic therapy with standard rehabilitation may lead to greater improvements in motor and functional outcomes compared with standard therapy alone. The observed effects likely reflect the contribution of increased training intensity and patient engagement introduced by the robotic component, supporting the feasibility of this approach as part of multidisciplinary rehabilitation.

The most pronounced statistically significant intergroup differences were observed in the primary outcome of the FMA-UE scale. In the intervention group, the increase in indicators exceeded the MCID, indicating a clinically significant improvement in the motor impairment of the upper limb.

This improvement was accompanied by systemic positive changes in daily activities (BI, FIM), motor activity (mWMFT, FAT) and subjectively perceived limitations in hand use (DASH). Notably, the intervention effect remained significant even after accounting for baseline differences between groups using covariance analysis, which strengthens the reliability of the findings.

Of particular note is the observed reduction in anxiety and depression (HADS) in patients receiving robotic treatment. This suggests that active engagement in controlled, targeted movements contributed to a positive emotional response and a sense of control over recovery. In this context, ReHand acts not only as a motor rehabilitation tool, but also as a component of comprehensive psychoemotional recovery after stroke.

An additional strength of the trial is the replicability of the effect across multiple scales with large effect sizes (*δ* ≥ 0.474), indicating that the changes were not random and were sustainable. Thus, the intervention demonstrated not only therapeutic efficacy but also broad applicability within a multidisciplinary rehabilitation practice.

Although the baseline scores for FIM and BI were significantly higher in the control group, this imbalance may have reduced the potential for further improvement in these patients due to a ceiling effect. To account for this, we performed an adjusted analysis using Quade rank ANCOVA, which confirmed that the observed improvements in FIM, BI, and HADS were not attributable to baseline imbalances, thereby strengthening the reliability of our findings.

### 4.2 Comparison with previous trials

The results of this RCT are consistent with the data of other published RCTs and meta-analyses confirming the effectiveness of robotic therapy for restoration of upper limb impairment after stroke.

The results obtained in this trial demonstrate a statistically and clinically significant improvement in upper limb impairment according to the FMA-UE scale, which is consistent with previously published data. In particular, a number of RCTs and systematic reviews have reported positive dynamics reaching clinically significant levels with the use of various forms of robotic therapy. In our study, the median increase in FMA-UE score was +16.5 points, with 91.7% of participants in the intervention group exceeding the MCID threshold of 5.25 points. By contrast, large meta-analyses reported mean FMA-UE gains of approximately +2–3 points (mean difference: +2.23; 95% CI 1.11–3.35) across 86 RCTs Other wearable robotic glove interventions reported mean improvements of +6.1 or +9.3 points (McCrea et al., 2021; Chien et al., 2020; Zhang et al., 2022; Tang et al., 2023; Wang et al., 2024).

An increase in BI scores indicates a growth in patient independence when performing basic daily activities, such as eating, personal hygiene, and moving. These changes are comparable with the results of other trials devoted to the use of a robotic system and confirm the effectiveness of this approach in the recovery period. In our study BI improved by +30 points, compared to typical increases of +12 to +20 points reported in previous robotic therapy studies (Bertani et al., 2017; Yang et al., 2023).

A decrease in NIHSS scores indicates a reduction of neurological deficit associated with the consequences of stroke, including motor, speech, and coordination functions. Similar dynamics have also been described in a number of clinical studies, where similar improvements were recorded upon completion of rehabilitation. Compared to this study, where patients with a mean NIHSS score of 2.7 showed modest cognitive improvements over time, our trial demonstrated a significantly greater neurological recovery, with a mean NIHSS reduction of −4.0, highlighting the superior effect of our intervention on global stroke severity (Girgenti et al., 2023).

Positive changes in the mWMFT scale, which assesses the quality of hand movements, and in the FAT, which is aimed at identifying the ability to perform functional actions, are consistent with the results of other trials. They reported that robotic therapy helps improve motor control and restore purposeful movements of the hand and forearm. In another study, the improvement in the WMFT score was +18.02 points, which is comparable to our result of +18.5, confirming a similar degree of upper limb motor function recovery. In other study, the FAT score improved by 2 points, whereas in our trial, the improvement was slightly higher at +2.3 points, indicating a comparable but slightly greater functional gain in upper limb use (Ma et al., 2022; Bovolenta et al., 2009).

The results on the FIM scale demonstrate an improvement in the overall functional independence of patients after robotic therapy. These data are consistent with the results presented in a number of studies, which also reported a significant increase in FIM upon completion of rehabilitation using robotic technologies. FIM score increased by +22.5, while meta-analyses usually indicate gains in the range of +8 to +15 points (Veerbeek et al., 2017; Kurosaki et al., 2022).

The reduction in DASH scores reflects a reduction in limitations in daily use of the upper limb. Similar positive changes have been described in a number of trials where the use of robotic therapy contributed to a reduction in functional limitations and an increase in patients’ participation in everyday activities. A DASH reduction of −28 points in our intervention group significantly exceeds the typical decrease of −10 to −15 points seen in earlier robotic rehabilitation study (Villafañe et al., 2018).

The reduction in HADS scores confirms the positive impact of intervention on anxiety-depressive symptoms in patients after a stroke. Similar results were presented in previously published trials where, after a course of rehabilitation, patients after stroke showed an improvement in their psycho-emotional state. Notably, HADS scores decreased by −13 points, reflecting improvements in psychoemotional status that are larger than those commonly documented and often secondary in earlier trials (den Brave et al., 2023; Cheng et al., 2018).

Thus, the combined results confirm the effectiveness of robotic therapy not only in restoring the upper limb, but also in increasing the patient’s functional independence, reducing neurological deficit and limitations in daily activities, and improving the psychoemotional state of patients in the post-stroke period.

It is important to note that most previous robotic rehabilitation trials have been conducted in patients with chronic stroke, when the window of spontaneous recovery and heightened neuroplasticity is largely closed. In contrast, the present study focused on individuals in the subacute phase, when the brain exhibits greater responsiveness to motor training intensity and experience-dependent plasticity, potentially amplifying the observed benefits.

### 4.3 Clinical significance and mechanisms of therapeutic action

The therapeutic effects achieved in this trial, in particular a significant improvement in the motor impairment of the paretic upper limb, functional independence and a decrease in psychoemotional disorders, can be explained by a number of complementary mechanisms.

Neuroplastic changes following stroke are strongly influenced by the patient’s level of active participation in therapy. Simple passive or repetitive movements without voluntary engagement induce only limited cortical reorganization. In contrast, intensive and meaningful task-oriented training elicits more robust and lasting neuroplastic adaptations.

The ReHand robotic therapy was designed to ensure a high degree of patient involvement. Patients actively initiated grasp–release movements and received continuous multimodal feedback, which enhanced motivation and maintained attention during repetitive motor practice. Such active engagement is critical for driving experience-dependent plasticity in the recovering brain.

Firstly, the ReHand robotic therapy promotes neuroplasticity by providing intensive, task-specific, and feedback-based motor training, a key mechanism of recovery after stroke. Repetitive stimulation of passive movements of the paretic limb, initiated by reproducing the movements of the patient’s healthy hand, provides proprioceptive afferentation. This, in turn, promotes the plastic properties of neuronal circuits, resulting in the creation of newly available networks and the strengthening of existing ones to generate motor output to the paretic hand. This is confirmed by a reliable statistically significant improvement in the FMA-UE scale, reflecting the restoration of isolated and synergistic movements, as well as in tests assessing the quality and functionality of hand movements (mWMFT and FAT).

Secondly, the ReHand system provides a repetition mode, in which the healthy hand sets the movement pattern, and the exoskeleton synchronously reproduces it on the paretic limb. This is achieved through built-in sensors and a motor transmission that is adjustable in speed and intensity. Although the system does not provide classic biological feedback, it ensures a high degree of patient involvement and allows consciously associating the movements of both hands, which promotes the activation of neural systems and motor learning.

Thirdly, the focus of training on performing actions that imitate everyday activity (grasping, releasing and holding) contributes to the transfer of skills to real life. This is reflected in the improvement of the BI and FIM scales. The principle of functional specificity (task-specific training) plays a key role in restoring the independence and activity of patients in everyday functioning.

Positive dynamics in anxiety and depression scores according to the HADS scale are associated with both an objective improvement in motor impairment and a subjective sense of progress, control and participation. Increased motivation and improved psycho-emotional state form a positive cycle of behavioral feedback, in which positive experience increases the patient’s participation in rehabilitation, thereby contributing to an increase in the therapeutic effect.

Thus, the obtained effects of ReHand therapy reflect its multi-level impact on neural connections, daily life activities, functional status of the upper limb and psycho-emotional state of patients, ensuring clinically significant stroke recovery.

In addition to its clinical benefits, ReHand therapy also demonstrates strong practical advantages for real-world implementation. From a practical perspective, the ReHand system offers a portable and relatively low-cost alternative to large stationary robotic platforms. Its user-friendly design minimizes the need for extensive clinician training and facilitates rapid integration into existing outpatient or home-based rehabilitation models. The device requires only basic instruction and can be operated by trained physiotherapists or rehabilitation nurses. This flexibility enhances its scalability and supports integration into multidisciplinary rehabilitation programs, particularly in resource-limited settings.

### 4.4 Strengths of the trial

This RCT has a number of significant methodological and organizational advantages, ensuring high internal validity, reproducibility and clinical relevance of its results.

First of all, the sample size (*n* = 120) exceeds most previously published RCTs in the field of post-stroke rehabilitation with robotic technologies. Such a numerical advantage provides sufficient statistical power not only to identify the primary outcomes, but also to reliably assess secondary effects, including psychoemotional aspects and functional independence.

Secondly, stratified randomization was used by two clinically significant criteria: the severity of motor deficit (according to the FMA-UE scale) and age. This allowed us to achieve a high degree of comparability of the groups at baseline and reduce the risk of confounding, which is of particular importance when analyzing data from a heterogeneous group of patients in the subacute period of stroke recovery.

Thirdly, the intervention lasted for 8 weeks, which exceeds the standard duration of many robotic programs (usually 4–6 weeks) and corresponds to modern concepts of time windows of plasticity and motor learning. A longer exposure to therapy not only contributes to the formation of stable motor patterns, but also provides a high chance of transferring functional improvements to everyday life, which is confirmed by the results on the BI and FIM scales.

Fourth, the intervention is based on the principle of a combined rehabilitation model: robotic therapy (ReHand) is supplemented by elements of a standard rehabilitation approach. Such integration ensures both additional efficiency and high clinical and practical applicability of the protocol, allowing it to be implemented in various medical institutions, including outpatient and rehabilitation centers.

The fifth significant aspect is strict adherence to a standardized protocol, including a clearly regulated load dosage, double registration of results and blind analysis. This minimizes the likelihood of systematic and random measurement errors.

Finally, internationally validated scales with high sensitivity to changes were used to assess the effectiveness. They cover different levels of functioning: motor (FMA-UE, mWMFT, FAT), functional-everyday (BI, FIM), neurological (NIHSS), behavioral (DASH), and psychoemotional (HADS). This integrated approach to assessment provides a multidimensional view of the impact of intervention on the patient.

In total, the trial is characterized by high methodological rigor, clinical relevance, and extrapolability, making it a valuable basis for formulating recommendations for the use of robotic therapy in stroke restorative medicine.

### 4.5 Limitations of the trial

Despite the high methodological rigor, this trial has a number of limitations that need to be considered when interpreting and generalizing the results.

First of all, the trial was conducted within a single clinical center, which limits external validity and reduces the ability to extrapolate data to more diverse populations.

Second, the trial design was single-blind, but patients were aware of the nature of intervention they were receiving. This could potentially affect subjective outcomes, especially those related to emotional state and self-assessment of functional improvement, despite the use of standardized scales (HADS, DASH).

The third major limitation of our study is the lack of patient follow-up. All assessments were conducted immediately after the 8-week intervention, which limits conclusions regarding the long-term sustainability of the observed improvements. To address this, a structured follow-up evaluation is planned at 3 and 6 months post-intervention as part of an ongoing extension of the study. These assessments will help determine the persistence of treatment effects over time and inform the need for maintenance or booster interventions.

The fourth limitation is related to technical characteristics of the robotic device. ReHand is a compact, portable system without an integrated biofeedback interface, movement visualization or automatic load adaptation. Although the simplicity of such design makes the device accessible and applicable in outpatient and home settings, it may limit the depth of sensorimotor integration and variability of training compared to more technologically advanced stationary platforms.

The study did not use neurophysiological and neuroimaging methods such as functional magnetic resonance imaging (fMRI), transcranial magnetic stimulation (TMS) and electromyography (EMG), which limits the ability to objectively assess the putative mechanisms of neuroplasticity underlying the observed clinical improvement.

The study did not stratify patients by lesion location, stroke territory, or hand dominance, which may influence the pattern of motor recovery. Future studies should address these factors to better understand their impact on the efficacy of robotic interventions.

Only patients with mild-to-moderate upper-limb impairment were included, which limits the generalizability of our findings to individuals with severe motor deficits. Further studies are required to evaluate the applicability of ReHand therapy in patients with more profound motor impairment.

Another limitation is that the intervention group received additional therapy time compared with the control group, as ReHand sessions were provided in addition to standard rehabilitation. Therefore, part of the observed improvement may be attributable to the increased total therapy dose or greater patient engagement. Future studies should include a time-matched control group or a three-arm design to better isolate the independent contribution of ReHand therapy.

These limitations do not diminish the significance of the results, but highlight the need for further multicenter, high-tech studies with a long-term follow-up.

### 4.6 Prospects and recommendations

Given the convincing results of this trial, further research efforts should be directed toward multicenter, technologically advanced studies with a prolonged observation to assess the sustainability of achieved effects.

Foremost, it is necessary to conduct multicenter randomized controlled trials with a larger sample, including patients at different stages of recovery (including acute and chronic phases). This will allow us to evaluate the versatility and adaptability of the method in more diverse clinical settings.

A key area of future trials should be the inclusion of a long-term follow-up (after 3, 6, 12 months) to assess the sustainability of therapeutic effect and the possibility of maintaining it without additional interventions. This is especially important for integrating the method into long-term rehabilitation programs.

The use of neurophysiological and neuroimaging methods also seems promising, which will allow an objective assessment of dynamics of corticospinal restructuring and confirm the hypothesis about the mechanisms of neuroplasticity stimulated by intervention.

From a technical point of view, further trials should be focused on the use of high-tech robotic systems, including visual and auditory feedback modules, adaptive algorithms based on artificial intelligence, error analysis systems and gamified task formats. The integration of such components can be crucial for increasing the effectiveness of intervention, its adaptation to home rehabilitation conditions and increasing patient adherence to long-term therapy.

Future work should focus on developing hybrid protocols that combine in-person and remote forms of rehabilitation, which is especially relevant in conditions of limited access to in-patient centers. Telerehabilitation approaches with real-time monitoring can improve the availability of therapy and ensure its continuity.

Thus, further development of robotic therapy should combine scientific validation, technical adaptation and clinical integration, which will ensure its inclusion in standardized algorithms of rehabilitation medicine.

## 5 Conclusion

The RCT showed that robotic therapy combined with a standard rehabilitation program leads to statistically significant improvement in motor impairment and functional use of the paretic upper limb in patients in the subacute phase of stroke. Participants in the intervention group demonstrated significantly better results on the FMA-UE, mWMFT, FAT scales, as well as on the functional independence indices (BI, FIM) compared to patients who received only standard treatment. This indicates greater improvements in coordinated movements, dexterity, and functional independence among patients who received combined therapy with ReHand and standard rehabilitation. Most patients in the intervention group achieved clinically meaningful gains in FMA-UE scores, suggesting that ReHand may serve as a feasible and beneficial adjunct to conventional therapy in subacute stroke rehabilitation.

In addition, the intervention was accompanied by a statistically significant decrease in anxiety and depression scores on the HADS scale, which emphasizes the important role of psychoemotional state during rehabilitation. The results indicate the need for a comprehensive and patient-oriented approach to rehabilitation after stroke.

The results confirm the clinical validity and therapeutic efficacy of portable robotic systems as part of multidisciplinary rehabilitation programs in the subacute period. Structured, targeted robotic therapy is a promising direction that can enhance traditional approaches and increase the availability of intensive motor stimulation under resource-limited conditions.

## Data Availability

The raw data supporting the conclusions of this article will be made available by the authors, without undue reservation.

## Glossary

RCT: Randomized Controlled Trial
FMA-UE: Fugl-Meyer Assessment for Upper Extremity
BI: Barthel Index
NIHSS: National Institutes of Health Stroke Scale
mWMFT: Modified Wolf Motor Function Test
FAT: Frenchay Arm Test
DASH: Disabilities of the Arm, Shoulder, and Hand
HADS: Hospital Anxiety and Depression Scale
FIM: Functional Independence Measure
rTMS: repetitive Transcranial Magnetic Stimulation
ARAT: Action Research Arm Test
WHO: World Health Organization
CT: Computed Tomography
MRI: Magnetic Resonance Imaging
MMSE: Mini-Mental State Examination
NYHA: New York Heart Association
MCID: minimal clinically important difference
CI: confidence intervals
SD: standard deviation
fMRI: functional magnetic resonance imaging
EMG: electromyography
TMS: transcranial magnetic stimulation
